# Impact of Leadership Style on Professional Ethics and Moral Courage of Perioperative Nurses: A Cross‐Sectional Study in Southern Iran

**DOI:** 10.1155/jonm/7099442

**Published:** 2026-02-18

**Authors:** Amirali Alizadeh, Erfan Rajabi, Bahador Pourdel, Fatemeh Vizeshfar

**Affiliations:** ^1^ Research Center for Evidence-Based Health Management, Maragheh University of Medical Sciences, Maragheh, Iran, mrgums.ac.ir; ^2^ Department of Operating Room, School of Allied Medical Sciences, Iran University of Medical Sciences, Tehran, Iran, iums.ac.ir; ^3^ Department of Nursing, School of Nursing and Midwifery, Shiraz University of Medical Sciences, Shiraz, Iran, sums.ac.ir

## Abstract

**Aim:**

This study aimed to investigate the association between leadership styles and professional ethics and moral courage among perioperative nurses in southern Iran.

**Background:**

Operating rooms are high‐pressure environments where leadership behaviors and ethical decision‐making are critical for safe surgical care.

**Methods:**

A cross‐sectional study was conducted with 251 perioperative nurses in Shiraz hospitals (June–August 2024). Standardized instruments were used: the Multifactor Leadership Questionnaire (MLQ), the Cadozier Professional Ethics Questionnaire, and the Sekerka Moral Courage Scale. Data were analyzed using Spearman’s correlation and robust multiple regression (bootstrapping) due to non‐normal data distribution.

**Results:**

Active leadership styles demonstrated strong positive correlations with both professional ethics (*r* = 0.852) and moral courage (*r* = 0.868). Robust multiple regression analysis confirmed that transformational leadership was the primary positive predictor of both outcomes (*β*_PE_ = 0.754; *β*_MC_ = 0.824). Transactional leadership was also a significant predictor of professional ethics (*β*_PE_ = 0.197) but not of moral courage (*p* = 0.330). The models explained a substantial portion of the variance for professional ethics (adjusted *R* ^2^ = 0.842) and moral courage (adjusted *R* ^2^ = 0.704).

**Conclusion:**

Active leadership is significantly associated with enhanced professional ethics and moral courage in perioperative nurses. Fostering transformational and transactional leadership is therefore recommended to improve the ethical climate in high‐pressure clinical settings.

## 1. Introduction

The operating room is critical in delivering medical and surgical treatments to patients. All surgical team members must demonstrate high productivity and coordination to succeed in this complex and demanding environment. Perioperative nurses, a vital team component, have essential responsibilities such as monitoring equipment, delivering patient care, and collaborating with other team members [1]. Given the intense work demands and the necessity for prompt and precise decisions, perioperative nurses are frequently placed in situations requiring not only technical skill but also ethical judgment and moral resolve [2]. These unique situational demands make perioperative nurses an especially important group for studying how clinical roles that require rapid judgment under pressure are shaped by the demanding ethical climate of the operating room.

Professional ethics are crucial for nurses’ performance. They involve adhering to professional principles and standards to ensure the delivery of high‐quality and fair medical services. This is especially important in demanding and high‐stress environments such as the operating room, as it can greatly impact team dynamics and clinical decisions [3]. Several studies have shown that a strong commitment to ethical standards, such as transparency, responsibility, and respect for the patient, improves treatment outcomes, patient satisfaction, and reduces medical errors [4, 5]. Moral courage is crucial in enhancing the quality of medical care in this particular setting. It enables nurses to uphold their ideals and values and make precise decisions when faced with moral responsibilities and complicated dilemmas [6]. This feature is especially important in the operating room, where the environmental demands and urgency of decisions are significant. Moral courage among perioperative nurses improves the quality of medical services and promotes a positive team environment. This is because nurses can handle moral dilemmas with greater assurance, leading to higher patient satisfaction and improved care standards [7].

In navigating such ethical challenges, leadership behavior within the operating room plays a critical role. According to Bass and Avolio’s Full Range Leadership Theory, leadership styles are categorized into transformational, transactional, and laissez‐faire. Each style has distinct implications not only for team coordination but also for ethical behavior and individual moral strength. Transformational leadership, with its emphasis on idealized influence and inspirational motivation, may empower nurses to act with ethical integrity and moral courage, particularly in ethically challenging situations. Transactional leadership focuses on reward and punishment, potentially reinforcing compliance with ethical standards but not necessarily promoting internalized moral values. Conversely, laissez‐faire leadership, marked by avoidance of decision‐making, may undermine ethical accountability and reduce moral engagement among staff. Understanding how these leadership behaviors interact with nurses’ ethical performance is especially critical in high‐stakes environments like the operating room [8].

Leadership behaviors have been suggested to influence both professional ethics and moral courage in healthcare settings. Studies indicate that supportive leadership may contribute to ethical climate and encourage staff to uphold moral values during decision‐making. In high‐pressure environments like the operating room, where ethical and clinical decisions often intersect, the style of leadership may play a role in shaping how nurses respond to complex ethical dilemmas.

According to the literature review, several studies have been conducted in this field. For example, the transformational leadership style has been shown to enhance professional commitment and resilience among nurses [9, 10]. Additionally, a study conducted among nurses in the hospital’s general wards found a statistically significant correlation between moral leadership and moral courage [11]. Previous research in Iran has also shown a link between leadership style and ethical concerns among nurses [11, 12].

In contrast to prior research that has mostly investigated leadership style, moral courage, and professional ethics in nonoperating room contexts, this study specifically focuses on the unique environment of the operating room. The objective of this study is to examine the relationship between leadership styles and two ethical constructs, professional ethics and moral courage, among perioperative nurses in operating room settings. Specifically, the study aims to assess how different leadership styles correlate with these ethical outcomes in a high‐pressure clinical environment. By focusing on perioperative nurses, the study addresses a gap in the existing literature, which has largely overlooked this specialized context.

## 2. Methods

### 2.1. Study Design

The cross‐sectional study took place from June to August 2024 in the operating rooms of hospitals affiliated with the Shiraz University of Medical Sciences, the largest city in southern Iran. The study was conducted in six major hospitals in Shiraz city, which are recognized as the primary surgical and specialized treatment facilities in the southern region of the country. The selected hospitals included both public and private institutions, all were under the academic and administrative oversight of the Shiraz University of Medical Sciences.

### 2.2. Study Participant

The study included all eligible perioperative nurses from the mentioned hospitals. Inclusion criteria were (1) being employed as a perioperative nurse, (2) having at least 1 year of clinical experience in the operating room, and (3) completing a written informed consent form. Exclusion criteria included (1) absence for any reason (such as leave or change of employer) during the study and (2) incomplete completion of the questionnaires. Upon receiving authorization to begin the study, the researchers visited the hospital and operating room and introduced themselves to relevant officials. They obtained a list of perioperative nurses and identified nurses who met the inclusion criteria.

### 2.3. Sample Size

The required sample size was estimated using Cochran’s formula for finite populations, assuming a 95% confidence level, a 5% margin of error, and a maximum variability proportion (*p* = 0.5). This calculation yielded a minimum target of 230 participants. To safeguard against potential data loss due to nonresponse or incomplete submissions, an anticipated attrition rate of 15% was incorporated into the sampling strategy. Accordingly, the sample size was inflated to 270 participants to ensure adequate statistical power. Of these, 251 nurses provided fully completed and analyzable responses and were included in the final analysis.

### 2.4. Study Processes and Tools

The questionnaire for data collection in this study was divided into four sections, and perioperative nurses completed it by self‐reporting.

The initial section included a demographic and clinical information questionnaire, covering age, gender, employment status, educational level, work experience, type of hospital (public or private), and usual work shift (morning, evening, or night). These questions were adapted from a relevant literature review and aligned with the study’s objectives. The researchers obtained the participants’ consent to take part in the study by explaining the investigation details to them while they were in the operating room during various work shifts.

The questionnaires were distributed directly by the primary researchers during various work shifts. Standardized instructions were provided to ensure that all participants understood the questionnaire items clearly and responded independently. Participants completed the questionnaires in a quiet area of the operating room and returned them immediately to the researchers upon completion.

The second part of the assessment involved a multifactor questionnaire that evaluates leadership styles. This questionnaire consists of 36 questions, which are divided into three subscales: transformational leadership (20 questions), transactional leadership (12 questions), and laissez‐faire leadership (4 questions). The questionnaire was developed by Bass and Avolio [13]. It uses a 5‐point Likert scale for scoring, ranging from 0 (indicating “not at all”) to 4 (indicating “regularly, but not always”). By combining the scores of each subscale and calculating their averages, the supervisor with the highest average score in a particular subscale is considered to have the dominant style in that area. The questionnaire is available in two separate formats: one for leaders and another for followers. It assesses an individual’s leadership style from both their perspective and that of their followers. For this study, the version designed for followers was used. Prior psychometric studies have consistently reported high internal consistency for the MLQ, with Cronbach’s alpha values ranging from 0.90 to 0.97. Confirmatory factor analysis in multiple contexts supports its construct validity, aligning with Bass and Avolio’s theoretical model [14–16].

The third part was the professional ethics questionnaire. This tool, developed by Cadozier, consists of 16 questions divided into eight subscales, with each subscale comprising 2 items: responsibility, honesty, justice and fairness, loyalty, superiority and competition, respect for others, sympathy with others, and respect for values and social norms [17]. The instrument uses a 5‐point Likert scale, with scores ranging from 1 (indicating very little) to 5 (indicating very lot). The potential scores range from 16 to 80. Scores between 16 and 32 indicate poor professional ethics, scores between 32 and 48 indicate average ethics, and scores above 48 indicate high professional ethics [18]. The psychometric properties of the Persian version were established by Jafari & Hanifi, who confirmed its content validity (CVI = 0.91 and CVR = 0.89), construct validity via a three‐factor structure explaining 63.15% of the total variance, and reliability with Cronbach’s alpha of 0.87 [19].

The fourth component includes the moral courage questionnaire developed by Sekerka et al. This instrument consists of 15 questions and five subscales, with each subscale measured by 3 items: moral agency, multiple values, threat endurances, beyond compliance, and moral goals. The scoring of this instrument is determined by a seven‐point Likert scale ranging from 1 (never true) to 7 (always true) (20, 21). The item scores in each dimension range from a minimum of 7 to a maximum of 21, and the overall score ranges from a minimum of 15 to a maximum of 105. Based on established scoring guidelines, total scores are categorized: 15–45 = low moral courage, 46–75 = moderate, and 76–105 = high courage [20, 21]. The questionnaire was translated into Persian by Mohammadi et al. in Iran, and its validity and reliability were verified with a coefficient of variation (CVI) of 81% and Cronbach’s alpha of 0.85 [22].

### 2.5. Study Analysis

All collected data were coded and entered into SPSS software version 16 for statistical analysis. The normality of the data distribution for the main research variables was assessed using the Kolmogorov–Smirnov test. The results indicated that the data did not follow a normal distribution (*p* < 0.001 for all variables), justifying the use of nonparametric statistical tests for subsequent analyses. Descriptive statistics, including mean, standard deviation, median, and frequency, were used to summarize demographic and clinical characteristics. For inferential analysis, Spearman’s correlation coefficient and Mann–Whitney *U* tests were employed to examine relationships between key variables and demographic factors. To identify predictors of professional ethics and moral courage, univariate linear regression analyses were initially performed. Variables with *p* values less than 0.2 in the univariate analyses were then included in the multivariate linear regression models to improve model stability and reduce the risk of excluding potentially relevant predictors. All statistical tests were two‐tailed, and a significance level of *p* < 0.05 was considered statistically significant.

### 2.6. Ethical Consideration

This research has been approved by the ethics committee of the Shiraz University of Medical Sciences with the ethics code IR.SUMS.NUMIMG.REC.1403.011. This study follows the guidelines for observational studies (STROBE). Before the study began, all participants were thoroughly informed about the study’s objectives. The perioperative nurses were told that their participation in the study is voluntary and will not affect their employment. Written informed consent was obtained from all participants prior to the study. To maintain confidentiality, each participant was given a code.

## 3. Results

### 3.1. Participant Characteristics

The study involved 251 individuals who completed the questionnaires, resulting in a response rate of 92.96%. As indicated in Table 1, the participants had an average age of 31.14 years (±8.52) and an average work experience of 6.80 years (±7.52). About 63.7% of the participants were female, and 76.5% were single. The majority of participants (68.1%) held a bachelor’s degree, and the majority (75.5%) were employed in a rotating shift system. Most of them were employed at government hospitals, making up 52.6% of the total, while the majority had an unofficial employment status of 68.1%.

**TABLE 1:** Demographic characteristics of participants (*N* = 251).

Variable	Frequency (%) and Mean (±SD)
Age (years)	31.14 (±8.52)
Sex	
Male	91 (36.3%)
Female	160 (63.7%)
Marital status	
Single	192 (76.5%)
Married	59 (23.5%)
Education	
Bachelor	171 (68.1%)
Master science	46 (18.3%)
PhD	34 (13.5%)
Shift	
Fix	61 (24.3%)
Rotational	190 (75.5%)
Hospital	
Public	132 (52.6%)
Private	119 (47.4%)
Job status	
Official	80 (31.9%)
Unofficial	171 (68.1%)
Experience (years)	6.80 (±7.52)

### 3.2. Mean Score of Variables and Their Dimensions

Among the different types of leadership styles, the transformational leadership style had the highest average score, while the Laissez‐faire leadership style had the lowest score. The average score for professional ethics was 57.64 (±9.22), and for moral courage, it was 57.71 (±7.70). Honesty had the highest average score among the different aspects of professional ethics, while superiority and competitiveness had the lowest scores. In terms of moral courage, moral agency received the highest marks, while moral goals received the lowest scores (Table 2).

**TABLE 2:** Mean score of main variables and their dimensions.

Variable	Mean	SD
Transformational Leadership Style	32.82	9.05
Transactional Leadership Style	21.63	7.79
Laissez‐faire Leadership Style	6.01	4.06
Professional ethics	57.64	9.22
Responsibility	7.74	1.56
Honesty	8.32	1.73
Justice	6.25	1.05
Loyalty	7.34	1.83
Superiority and Competitiveness	5.91	2.32
Respect for others	6.80	1.68
Sympathy	7.67	1.70
Respect for social values	7.59	1.95
Moral courage	57.71	7.70
Moral agency	12.62	1.66
Multiple values	11.40	1.90
Threat endurance	11.77	2.14
Beyond compliance	11.27	2.40
Moral goals	10.63	1.80

### 3.3. Mean Score of Variables According to Demographic Information

**FIGURE 1:**
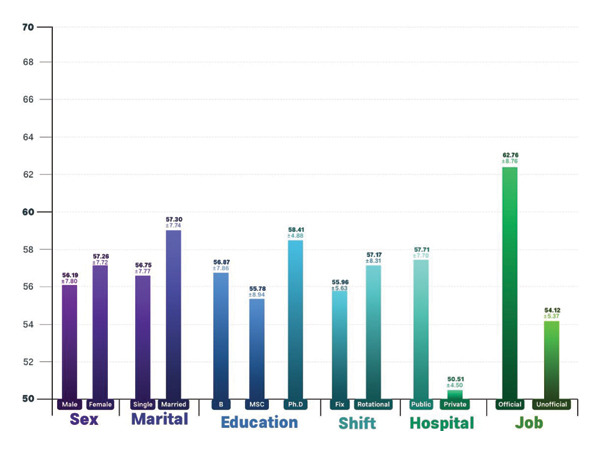
Demographic variables according to moral courage.

**FIGURE 2:**
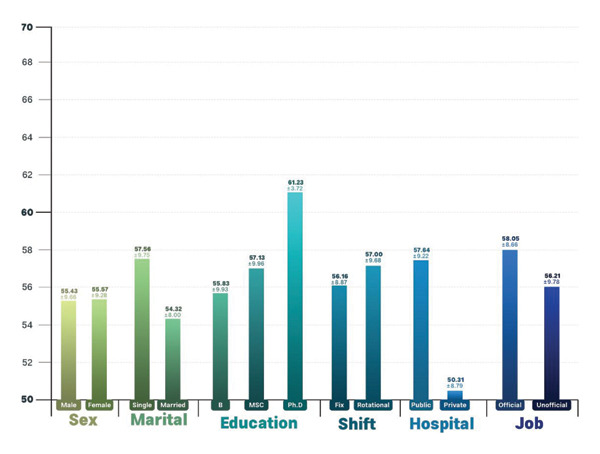
Demographic variables according to professional ethics.

The data from Figures 1 and 2 indicate that female participants scored higher in professional ethics and moral courage compared to male participants. Furthermore, individuals with a doctoral degree performed better in both professional ethics and moral courage than those with a bachelor’s or master’s degree. Nurses working in rotating shifts had higher scores in professional ethics than those working fixed shifts. Additionally, employees in public hospitals demonstrated stronger professional ethics and moral courage than those in private hospitals. Finally, official employees scored higher in both professional ethics and moral courage compared to informal employees.

### 3.4. Correlation of Variables

The results of Spearman’s correlation analysis (Table 3) indicated that both transformational (*r* = 0.852, *p* < 0.001) and transactional (*r* = 0.819, *p* < 0.001) leadership styles were positively correlated with professional ethics. Similar results were observed for moral courage; both transformational (*r* = 0.868, *p* < 0.001) and transactional (*r* = 0.772, *p* < 0.001) leadership styles had a significant positive relationship with this variable. In contrast, the laissez‐faire leadership style exhibited a significant negative correlation with moral courage (*r* = −0.127, *p* = 0.045). Additionally, a positive correlation was found between professional ethics and moral courage (*r* = 0.774, *p* < 0.001).

**TABLE 3:** Correlation analysis between research variables.

Variable	TRF^a^	TRA^a^	LF^a^	PE^a^	MC^a^
TRF	1				
TRA	*r* = 0.766^∗∗^ *p* < 0.001	1			
LF	*r* = −0.237^∗∗^ *p* < 0.001	*r* = 0.211^∗∗^ *p* < 0.001	1		
PE	*r* = 0.852^∗∗^ *p* < 0.001	*r* = 0.819^∗∗^ *p* < 0.001	*R* = −0.096 *p* = 0.128	1	
MC	*r* = 0.868^∗∗^ *p* < 0.001	*r* = 0.772^∗∗^ *p* < 0.001	*R* = −0.127^∗^ *p* = 0.045	*r* = 0.774^∗∗^ *p* < 0.001	1

*Note:* TRF: transformational leadership, TRA: transactional leadership, LF: laissez‐faire leadership.

Abbreviations: MC = moral courage, PE = professional ethics.

^a^Spearman’s correlation coefficient.

^∗^Correlation is significant at level 0.05 (2‐tailed).

^∗∗^Correlation is significant at level 0.01 (2‐tailed).

Analysis of the relationship between demographic variables and leadership subscales revealed that age was positively and significantly correlated with all three leadership styles. Furthermore, the Kruskal–Wallis test indicated a significant difference in the scores of all three leadership styles based on the participants’ level of education (*p* < 0.005).

### 3.5. Regression Analysis

Multiple linear regression models were employed to identify the predictive relationships between leadership styles (transformational and transactional) and the two outcome variables: professional ethics and moral courage.

#### 3.5.1. Regression Assumption Check

Prior to interpreting the final models, the regression assumptions were rigorously checked:

• Multicollinearity: The variance inflation factor (VIF) for both transformational and transactional leadership styles was calculated at 3.785 (tolerance = 0.264), indicating no serious multicollinearity issues.
• Normality of residuals: The Kolmogorov–Smirnov test performed on the residuals of both models indicated a significant deviation from a normal distribution (*p* < 0.001).
• Robust method: Given the violation of the normality assumption, bootstrapping (based on 1000 resampling draws) was utilized to derive robust standard errors and confidence intervals, thus ensuring the validity of the statistical inferences.

#### 3.5.2. Prediction of Professional Ethics

The first model, predicting professional ethics, was statistically significant (*F* (2, 248) = 666.744, *p* < 0.001) and accounted for a substantial amount of variance (adjusted *R* ^2^ = 0.842). The overall effect size for the model was large (Cohen’s *f* ^2^ = 5.37).

As presented in Table 4, both active leadership styles were significant positive predictors: transformational leadership (beta = 0.754, *p* < 0.001) was the strongest predictor, and transactional leadership was also a significant predictor (beta = 0.197, *p* < 0.001).

**TABLE 4:** Robust multiple regression analysis predicting professional ethics (N = 251).

Predictors	Unstandardized B	Bootstrap SE	Standardized *β*	*t*	*p*‐value	95% confidence interval (B)	
						Lower	Upper
(Constant)	8.473	2.025		4.252	< 0.001	4.763	12.602
Transformational	0.768	0.048	0.754	19.222	< 0.001	0.672	0.863
Transactional	0.234	0.054	0.197	5.028	< 0.001	0.129	0.344

*Note:* Overall model statistics: *F* (2, 248) = 666.744, *p* < 0.001; adjusted *R* ^2^ = 0.842. The model shows no serious multicollinearity (VIF < 5). Bootstrap results are based on 1000 samples.

#### 3.5.3. Prediction of Moral Courage

The second model, predicting moral courage, was also significant (*F* (2, 248) = 298.486, *p* < 0.001) and explained a large proportion of variance (adjusted *R* ^2^ = 0.704). The effect size for this model was also large (*f* ^2^ = 2.39).

As shown in Table 5, transformational leadership was the sole significant predictor, demonstrating a very strong positive association (beta = 0.824, *p* < 0.001). Transactional leadership did not reach statistical significance in this model (beta = 0.053, *p* = 0.330). The laissez‐faire leadership style and all demographic variables were excluded from the final robust models due to lack of significance.

**TABLE 5:** Robust multiple regression analysis predicting moral courage (*N* = 251).

Predictors	Unstandardized B	Bootstrap SE	Standardized *β*	*t*	*p*‐value	95% confidence interval (B)	
						Lower	Upper
(Constant)	17.191	1.987		7.961	< 0.001	13.033	20.916
Transformational	0.701	0.069	0.824	14.746	< 0.001	0.572	0.839
Transactional	0.052	0.070	0.053	0.976	0.330	−0.086	0.186

*Note:* Overall model statistics: *F* (2, 248) = 298.486, *p* < 0.001; adjusted *R* ^2^ = 0.704. The model shows no serious multicollinearity (VIF < 5). Bootstrap results are based on 1000 samples.

Demographic variables (including age, gender, education, shift work, and experience) were tested in initial models but were not significant predictors of either outcome in the final robust multivariate analyses and were excluded for model parsimony.

## 4. Discussion

The primary finding of this investigation is the strong, positive, and significant association of active leadership styles with the two ethical outcomes. Specifically, transformational leadership proved to be the most consistent and powerful predictor for both professional ethics and moral courage, while transactional leadership was significantly associated only with professional ethics. These results are particularly significant when interpreted within the high‐stakes environment of the operating room.

### 4.1. Interpretation of Key Findings Within the Operating Room Context

The primary finding of this investigation is the strong, positive, and significant association of both transformational and transactional leadership styles with the professional ethics and moral courage of nurses. These results are particularly significant when interpreted within the high‐stakes environment of the operating room. The study’s findings align coherently with Bass’s Full Range Leadership Model, which situates leadership styles on a continuum from passive (laissez‐faire) to highly active and effective (transformational).

Transformational leadership, as the strongest correlate, is likely linked to higher ethical and moral conduct by fostering a climate of psychological safety and shared values amidst the intense pressures of surgical procedures. In an environment where split‐second decisions are critical, behaviors such as articulating a compelling vision (inspirational motivation) and acting as ethical role models (idealized influence) are crucial. They encourage nurses to transcend mere task completion and commit to a collective goal of patient safety, a finding consistent with research on improving clinical performance and team dynamics in high‐pressure medical settings [23–25].

Furthermore, the affirmation that this style is associated with stronger moral courage and professional ethics is especially relevant for operating room nurses, who frequently face complex ethical dilemmas. Similarly, the significant positive relationship observed for transactional leadership is highly applicable to the structured nature of the operating room. By clarifying roles, monitoring for errors, and providing clear feedback, transactional leaders create a predictable and accountable environment. This structure is vital for minimizing risks in surgical settings and can bolster both the ethical framework and the confidence required for moral courage.

While our findings strongly support the positive role of active leadership, it is important to acknowledge that some prior research outside the OR context has reported mixed or weaker associations, particularly for transactional leadership. For instance, some studies suggest that an over‐reliance on transactional rewards might undermine intrinsic moral motivation over time. However, our results suggest that in the highly regulated and procedural environment of the OR, the clarity and accountability provided by transactional behavior may be uniquely beneficial and complementary to transformational inspiration, rather than detrimental.

Conversely, the significant negative correlation between laissez‐faire leadership and moral courage highlights a critical risk in this specific context. A passive‐avoidant leadership style in the operating room represents a dangerous vacuum, leading to role ambiguity and increased stress. This can suppress the willingness of nurses to speak up about potential errors or ethical breaches, thereby directly being linked to compromised patient safety and quality of care [26, 27]. Interestingly, our robust regression model did not find a significant independent association between laissez‐faire leadership and professional ethics, suggesting that its negative impact may be more pronounced on the courage to act rather than the underlying ethical principles themselves.

### 4.2. Interpretation of Mean Scores in the Perioperative Context

To contextualize the participants’ standing, their mean scores were examined. The mean score for moral courage (57.71) falls within the “moderate” range. This moderate level may be a reflection of the unique challenges of the operating room, where hierarchical structures and the need for rapid, unquestioning execution can create significant organizational barriers. These factors can inhibit the translation of a nurse’s internal moral reasoning into overt action, a difficulty emphasized in studies on moral decision‐making in stressful environments [28–32].

In contrast, the mean score for professional ethics (57.64) is situated in the “high” range, indicating a strong self‐reported adherence to ethical principles. An analysis of the ethics subscales revealed that “honesty” received the highest mean score, while “superiority and competitiveness” scored the lowest. This suggests that while operating room nurses prioritize integrity, the intense, team‐based nature of their work may not foster a culture of individual competition. This resonates with findings by Teymoori et al., who identified structural limitations to individual career advancement within operating room nursing in Iran [1]. The strong positive correlation between professional ethics and moral courage (*r* = 0.774) is also consistent with prior research, suggesting that a strong ethical foundation is a prerequisite for courageous action in the demanding perioperative setting [7, 31].

### 4.3. Implications for Nursing Management

The results of this study offer clear, evidence‐based implications for nursing management, which are now fully aligned with the empirical data. Given the potent, positive association of active leadership styles, it is recommended that hospital administrators and nurse managers

1. Develop and promote active leadership behaviors: Implement targeted training programs designed to cultivate transformational leadership competencies (e.g., articulating a vision, coaching) and effective transactional skills (e.g., clarifying expectations, providing contingent rewards).
2. Mitigate laissez‐faire leadership: Actively identify and address passive‐avoidant leadership through performance management, feedback mechanisms, and leadership coaching, given its detrimental association with moral courage.
3. Cultivate a supportive ethical milieu: Leverage the demonstrated link between positive leadership and ethical outcomes by fostering an organizational culture where ethical discourse is encouraged, and moral courage is explicitly supported and valued.

### 4.4. Limitations and Directions for Future Research

The strengths of this study include the use of standardized, validated instruments and its focus on the critical environment of the operating room. Nonetheless, certain limitations must be acknowledged. The cross‐sectional design precludes causal inferences, limiting our findings to associations rather than definitive effects. The study’s confinement to a single city limits the generalizability of the findings. Finally, the reliance on self‐report measures carries an inherent risk of social desirability bias.

Future research should utilize longitudinal or experimental designs to better establish causal relationships. Specifically, future studies utilizing structural equation modeling (SEM) or path analysis are recommended to test theoretical frameworks involving potential mediating or moderating variables. Replicating the study across diverse geographical and cultural contexts would enhance external validity. Furthermore, employing mixed‐methods research that integrates quantitative surveys with qualitative interviews could offer a richer, more nuanced understanding of nurses’ lived experiences with leadership and ethical dilemmas.

## 5. Conclusion

This study provides compelling evidence that specific, active leadership styles are profoundly and positively associated with professional ethics and moral courage among perioperative nurses. The findings demonstrate that transformational leadership is the pivotal predictor for both outcomes, with transactional leadership also being significantly associated with professional ethics. Consequently, it is strongly recommended that healthcare organizations invest in the development of active and supportive leadership to cultivate a robust ethical climate, which is associated with improving the quality of patient care and empowering nurses to act with moral conviction.

## Author Contributions

Amirali Alizadeh: conceptualization, data curation, methodology, writing–original draft, and writing–review and editing. Erfan Rajabi: administration, conceptualization, and supervision. Bahador Pourdel: Formal analysis, visualization, and writing–review and editing. Fatemeh Vizeshfar: supervision, data curation, and investigation.

## Funding

This work was supported by the Vice‐chancellors for Research Affairs at the Shiraz University of Medical Sciences, 29722.

## Disclosure

The funding body did not play any role in the design of the study, the collection, analysis, or interpretation of data, or in the writing of the manuscript.

## Conflicts of Interest

The authors declare no conflicts of interest.

## Data Availability

The data that support the findings of this study are available on request from the corresponding author. The data are not publicly available due to privacy or ethical restrictions.
